# The Relationship between Multiple Myeloma with Renal Failure and Metastatic Calcification

**DOI:** 10.1155/2018/7819792

**Published:** 2018-06-20

**Authors:** Takanori Fukuta, Takayuki Tanaka, Yoshinori Hashimoto, Hiromi Omura

**Affiliations:** Department of Hematology, Tottori Prefectural Central Hospital, Tottori, Japan

## Abstract

While cases of multiple myeloma (MM) with metastatic calcification have been reported, the mechanisms for this calcification have yet to be explained. We observed a case of MM in a patient with end-stage renal failure who developed vascular and pulmonary calcification. A 51-year-old male was diagnosed with Bence-Jones type MM and required maintenance hemodialysis. He was treated with bortezomib-dexamethasone, vincristine-doxorubicin-dexamethasone, the M2 protocol, and lenalidomide-dexamethasone (Rd) therapy. During the sixth cycle of Rd therapy, he complained of pain in both lower legs. Well-demarcated ulcers with severe pain had developed on the right lower leg, both exterior thighs, and penis. We found that the patient's serum intact parathyroid hormone level was elevated, while it had previously been permissively controlled. Computed tomography scan showed widespread centrilobular opacities of the bilateral lungs and high-density lesions along small blood vessels in the trunk and all four extremities. Histological calcifications were identified in small blood vessels and the alveolar walls. The risk of metastatic calcification in MM appears to be associated with renal failure, but not with MM itself.

## 1. Introduction

Multiple myeloma (MM) is a clonal plasma cell proliferative disorder with symptoms related to bone marrow infiltration, impaired hematopoiesis, or end-organ damage, which ultimately leads to renal failure, bone lesions, and hypercalcemia.

Metastatic calcification is the deposition of calcium salts in systemic organs. Calcific uremic arteriolopathy (CUA), a type of metastatic calcification, is a rare condition characterized by cutaneous artery calcinosis, leading to skin ischemia and ulceration. The term “calciphylaxis” is also used to describe such lesions, but it was originally used to describe hypersensitivity [1].

Cases of MM with metastatic calcification have been previously reported, but the mechanism for this calcification is unclear. Here we describe a patient with MM and end-stage renal failure who developed vascular and pulmonary calcification, and we examine the relationship between MM and calcification.

## 2. Case Presentation

**Figure 1:**
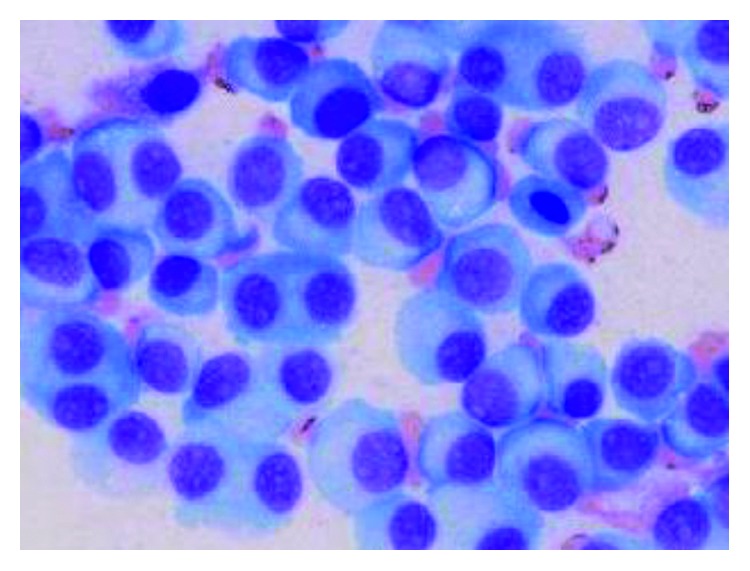
Morphology of the plasma cells in a bone marrow smear (May–Giemsa staining).

A 51-year-old male was referred to our hospital because of a three-month history of gradually progressing renal failure. During his first hospitalization, he complained of lumbar pain. On physical examination, he had conjunctival pallor and severe percussion tenderness of his back. No skin lesions or neurological deficits were seen. Laboratory test results were as follows: hemoglobin, 8.7 g/dL; creatinine, 7.01 mg/dL; total protein, 7.4 g/dL; albumin, 3.2 g/dL; calcium, 14.8 mg/dL; phosphate, 6.2 mg/dL; beta-2-microglobulin, 27.9 mg/L; IgG, 341 mg/dL; IgA, 21 mg/dL; IgM, 18 mg/dL; free kappa light chain, 99,900 mg/L; and free lambda light chain, 9.7 mg/L. Chest X-ray results were normal. Computed tomography (CT) showed vertebral compression fractures of Th8 and L1 and bilateral pleural effusions without calcified lesions. Urine immunoelectrophoresis showed a positive result for the Bence-Jones protein. Bone marrow aspiration revealed plasma cell proliferation (65% of total nucleated cells, Figure 1) with expression of CD38 and CD56, absence of CD19 and CD20, and an MIB-1 labeling index of 25%. Chromosomal analysis of the bone marrow by G-banding showed a normal 46,XY karyotype, but fluorescence in situ hybridization revealed the abnormalities del(13q) and *t*(4;14). He was diagnosed with Bence-Jones protein type MM (stage III according to the International Staging System, and stage IIIB according to the Durie–Salmon classification system).

We began treatment with intravenous fluids and intramuscular injections of calcitonin to treat the severe hypercalcemia. Simultaneously, he received bortezomib-dexamethasone (Bd) therapy (subcutaneous injection of 1.3 mg/(m^2^·day) bortezomib plus 20 mg/day dexamethasone orally on days 1, 4, 8, and 11). Unexpectedly, he experienced severe acute heart failure on day 8, and temporarily required the support of a mechanical ventilator. Bd therapy was discontinued during the first treatment cycle. Because renal function had not improved, maintenance hemodialysis was initiated. Subsequently, we continued MM treatment with two cycles of vincristine-doxorubicin-dexamethasone (0.4 mg/body of vincristine and 9 mg/m^2^ of doxorubicin on days 1 to 4; and 40 mg/body of dexamethasone on days 1 to 4, 9 to 12, and 17 to 20 of a 28-day cycle) and the M2 protocol (multiple chemotherapeutic agents, not including proteasome inhibitors), followed by lenalidomide-dexamethasone (Rd) therapy (5 mg/day lenalidomide on days 1 to 21 plus 20 mg/body dexamethasone on days 1, 8, 15, 22 of a 28-day cycle).

About four months after starting Rd therapy, the patient suffered from myoclonus-like movement of the lower extremities. During the sixth cycle of Rd therapy, he complained of pain in both lower legs, but did not have skin lesions or tenderness. He had been taking loxoprofen, fentanyl (patch and buccal tablet), mecobalamin, ferrous fumarate, lansoprazole, amlodipine, furosemide, alfacalcidol, and darbepoetin alfa. It was unlikely that his pain was drug-induced.

**Figure 2:**
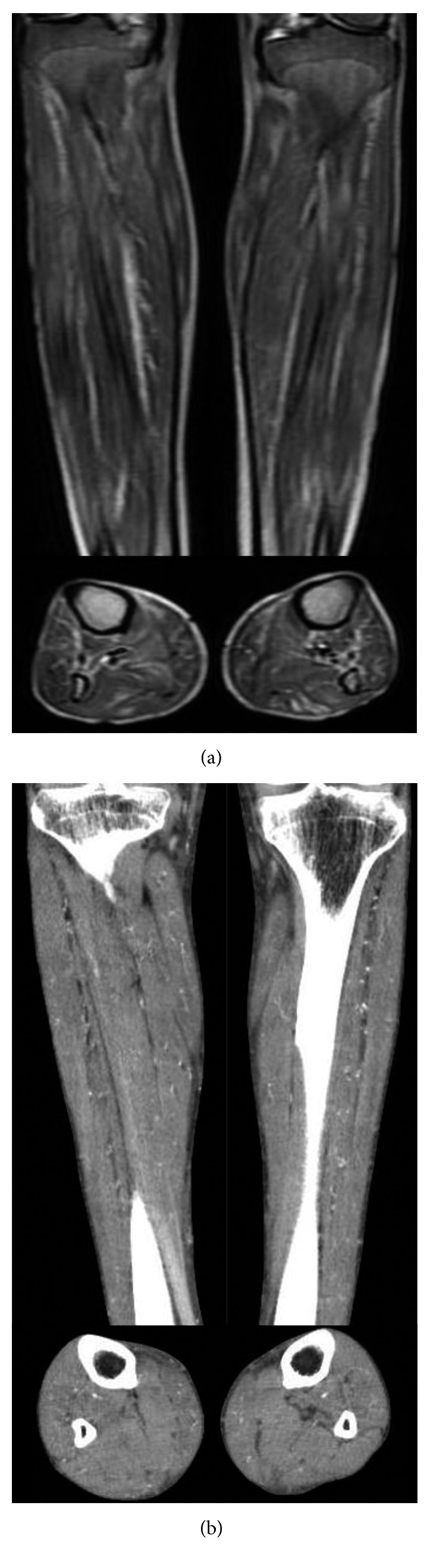
MRI/CT image of lower legs. (a) STIR (short tau inversion recovery) magnetic resonance image showing high-signal intensity areas in the lower leg muscles. These lesions had high DWI signals and normal ADC values. The muscle structure was intact. (b) Noncontrast enhanced computed tomography showing high density areas along the vessels.

**Figure 3:**
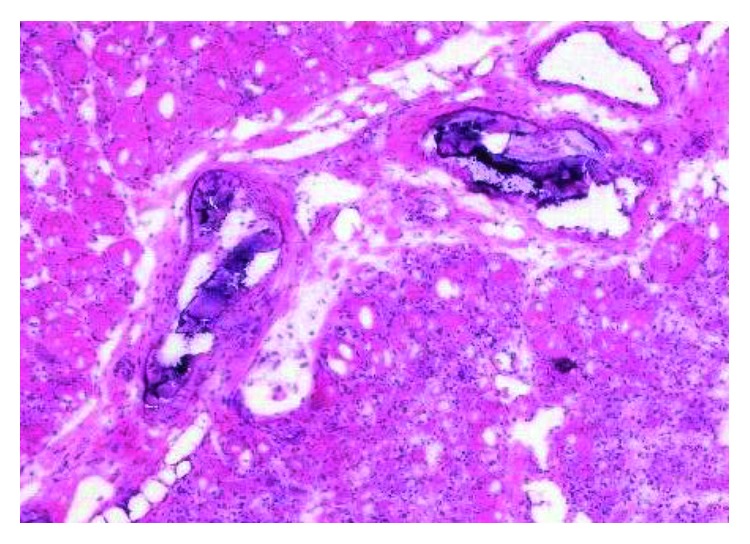
Muscle biopsy of the right anterior tibial muscle (hematoxylin-eosin staining). Calcifications are observed in the vascular walls among the muscular bundles. Muscle fibers are slightly basophilic, and myophagia is also observed.

**Figure 4:**
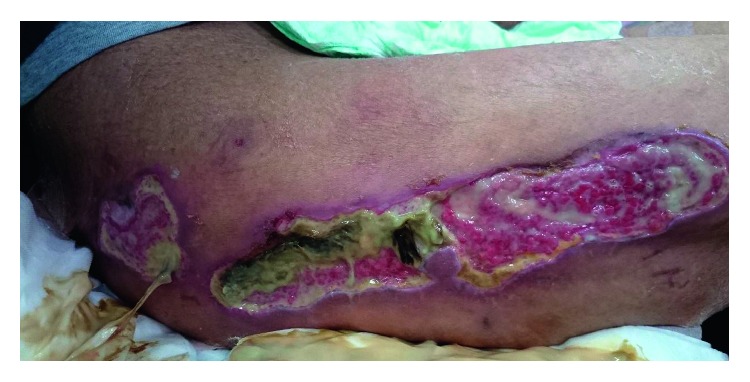
Refractory ischemic skin ulcers of the right exterior thigh due to vascular calcification. This wound later reached the fascia.

The patient's serum creatinine kinase level was elevated to 1,268 U/L. Diffusion-weighted and short tau inversion recovery magnetic resonance imaging revealed diffuse high signal intensity in the crural muscles (Figure 2(a)). A muscle biopsy was performed on the right tibial anterior muscles (Figure 3) and 40 mg/day prednisolone was prescribed by a neurologist because of suspected polymyositis/dermatomyositis. However, typical pathologic findings of polymyositis/dermatomyositis, like lymphocyte infiltration around muscle fibers, were absent and vessel calcification was noted. Prednisolone was ineffective against his symptoms. During steroid administration, well-demarcated ulcers developed on the right lower leg, both exterior thighs, and the penis. These ulcers gradually worsened (Figure 4) and the patient experienced severe pain, especially during dialysis or exercise. He could not continue dialysis because of this exacerbation of pain. Moreover, muscle atrophy of his lower limbs impaired his daily activities. The administration of 40 mg/day prednisolone was continued for 42 days and was then stopped on the 84th day after tapering. The patient's serum intact parathyroid hormone (PTH) level was 429 pg/mL, while previously it was permissively controlled within the range of 140–250 pg/mL. Before dialysis, his levels of serum albumin, calcium, and phosphate were 4.0 g/dL, 7.4 mg/dL, and 7.9 mg/dL, respectively. He was diagnosed with secondary hyperparathyroidism (HPT). We could not exclude a relationship between MM and HPT, although his free light chain ratio was decreasing.

**Figure 5:**
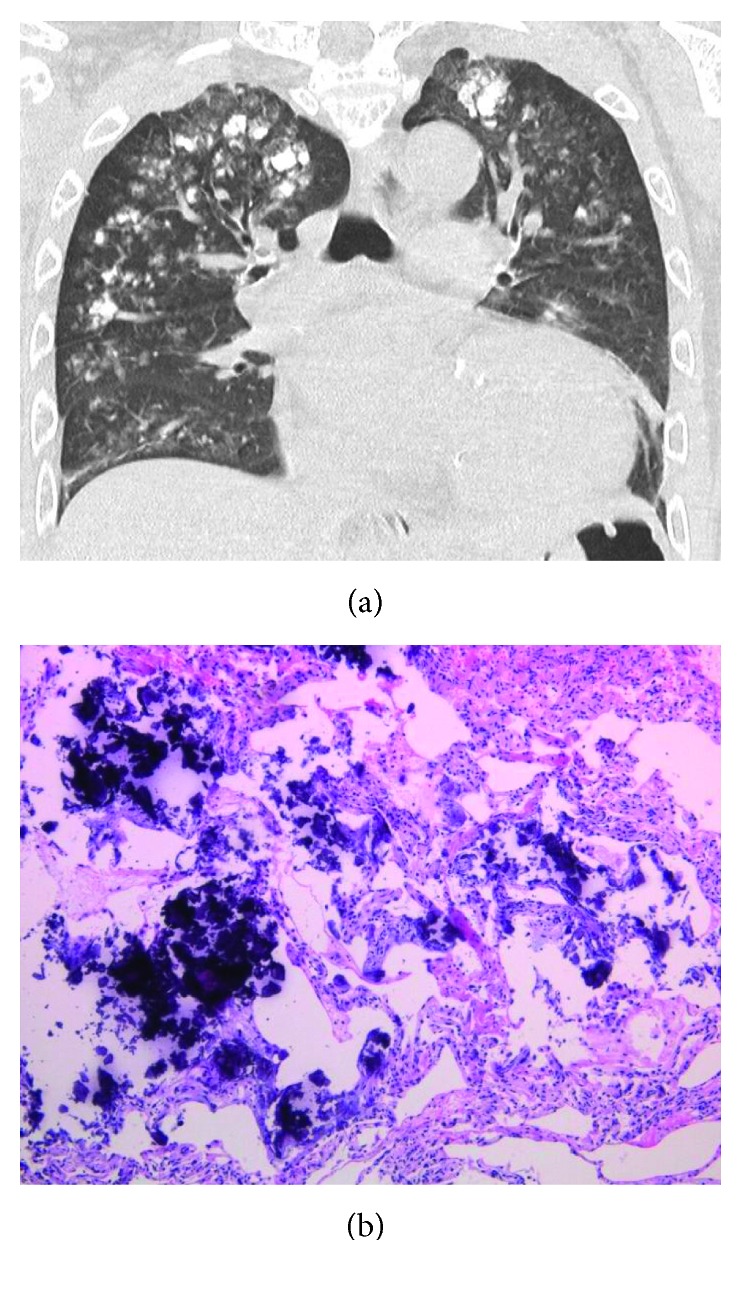
(a) Computed tomography of the lungs. Calcifications were observed in both the lungs, especially in the upper fields. (b) Transbronchial lung biopsy specimen (hematoxylin-eosin staining). Basophilic fine calcific substances were observed along the alveolar wall.

**Figure 6:**
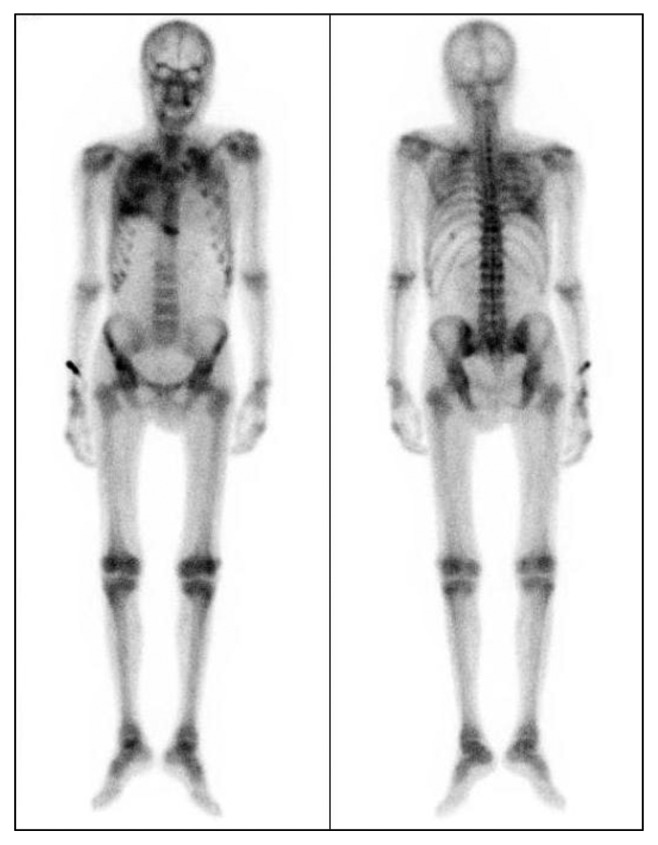
^99m^Tc-hydroxymethylene diphosphonate scintigraphy. Accumulation in both of the upper lung fields was detected.

CT showed widespread centrilobular opacities of both lungs (Figure 5(a)) and high-density lesions along small blood vessels in the trunk and extremities (Figure 2(b)), but the mediastinum, abdominal organs, and large vessels like the thoracic and abdominal aorta were intact. A pulmonary function test demonstrated restrictive impairment with reduced diffusion capacity: the predicted forced vital capacity was 48.6%, the forced expiratory volume of the first breath was 87.9%, and the predicted diffusing capacity of lung for carbon monoxide was 67.1%. ^99m^Tc-hydroxymethylene diphosphonate scintigraphy revealed abnormal diffuse accumulation in both upper lung fields (Figure 6). Echography revealed no enlargement of the parathyroid glands.

Next, a transbronchial lung biopsy was performed, and microscopy confirmed the presence of calcifications of the alveolar walls (Figure 5(b)) and of a small vessel in the right anterior tibial muscle (Figure 3). However, pathological calcification was absent from the right exterior thigh ulcer. Healing of the skin biopsy wound was delayed.

The patient was ultimately diagnosed with muscle and skin ischemia from CUA. He was treated with cinacalcet, and his intact PTH levels fell to a normal range. He underwent four additional cycles of Rd therapy, but ulcer infections occurred repeatedly in both thighs, occasionally progressing to sepsis. He has since been monitored closely for MM, without treatment, for four months. Meanwhile, the ulcers have achieved epithelialization after topical treatment, but his serum free light chain ratio level increased from 290 to 1532. He is currently undergoing Pd therapy (2 mg/day pomalidomide on days 1 to 21, plus 4 mg/day dexamethasone on days 1, 8, 15, and 22 of a 28-day cycle) without any adverse events. Since severe heart failure occurred during the combined regimen with bortezomib, we have avoided administering proteasome inhibitors. His disease is stable according to the International Myeloma Working Group criteria.

## 3. Discussion

In this case, metastatic calcification occurred in an MM patient with end-stage renal failure and secondary HPT. Melosalgia triggered the diagnosis of CUA. Histological calcifications were observed in small blood vessels and the alveolar walls. Cutaneous ulcers were found symmetrically in our patient, on both lower limbs and the penis, which were accompanied by strong pain exacerbated by dialysis and exertion. This symptom is consistent with ischemia: dialysis reduces the circulating plasma volume, while exertion increases oxygen demand. Clinicians should avoid biopsy for the definite diagnosis of suspected CUA based on clinical presentations, because such lesions may exhibit delayed wound healing. CUA causes a high mortality rate due to sepsis from wound infection [2]. The myoclonus-like movement of the lower extremities of our patient might have been another consequence of ischemia.

The mineral and bone metabolism of patients with renal failure should be controlled to improve prognosis. Recently, the term “chronic kidney disease-mineral and bone disorder” has been used for the condition traditionally called renal osteodystrophy. Complex abnormalities of calcium, phosphate, and PTH are all part of chronic kidney disease-mineral and bone disorder.

The relationship between MM and HPT is unclear. Hussain et al. described 30 cases of MM with primary HPT and reported that this condition is more common in females, its effects are observed in various types of MM immunoglobulin chains, and it does not coincide with the appearance of HPT. Unfortunately, the frequency of renal failure in these patients is not currently available [3]. Our hypothesis is that the main risk factor for HPT is not MM, but renal failure. Secondary HPT is common within dialysis populations. For example, Hedgeman et al. reported that the prevalence of secondary HPT within dialysis populations ranges from about 30 to 50% [4].

Interestingly, our patient developed metastatic pulmonary calcification, depositions of calcium in the pulmonary parenchyma, and pathologically identified calcifications of the alveolar walls, but not of lung small vessels. CT images showed a relatively strong deposition of calcium in the upper lung zone, which is typical of metastatic pulmonary calcification. It has been reported that the ventilation-perfusion ratio of the lung apex is higher than that of the base; therefore, the partial pressure of carbon dioxide in the artery is low and its pH is high. This environment appears to facilitate tissue calcification [5]. These lung lesions often do not cause respiratory failure and they are difficult to detect by chest radiography [5]. Kaltreider et al. found just 13 cases of interstitial pulmonary calcification in a series of 7,221 autopsies [6]. In contrast, metastatic pulmonary calcification was observed in 60% (9/15) [7] to 75% (42/56) [8] of chronic dialysis patients in an autopsy series. Chronic dialysis thus appears to carry a high risk of lung calcification.

To clarify the relationship between MM and metastatic calcification, PubMed was searched using the terms “multiple myeloma,” and “metastatic calcification,” and we then added other appropriate articles published between 1980 and 2015.  Table 1 shows data from 29 MM patients with metastatic calcification or CUA. Twenty-four of the patients (92%, excluding three with unclear renal function) presented renal insufficiency, and 26 (90%) developed hypercalcemia. Calcification of both the lungs and vessels were confirmed in eight patients (28%). PTH values were available in few cases. The type of immunoglobulin light and heavy chains observed were not uniform. These previous reports indicate that myeloma does not seem to have a primary role in metastatic calcification. We hypothesize that renal failure, not only in patients requiring dialysis, is a fundamental cause of calcinosis in MM patients.

The risk of metastatic calcification in MM appears to have a strong relationship with renal failure, but not with MM itself. Metastatic calcification, such as CUA and metastatic pulmonary calcification, is rare complication in MM patients, even in those with renal failure. However, clinicians should be aware of this condition, because it can induce organ injury or lethal outcomes.

## Figures and Tables

**Table 1:** Data for patients with multiple myeloma plus lung or vessel calcifications.

Age	Sex	Immunoglobulin chain	Cr (mg/dL)	Corrected Ca (mg/dL)	*P* (mg/dL)	Intact PTH (pg/mL)	Lung lesions^∗∗^	Pathological vessel calcifications	Reference
45	M	Lambda	2.6	12.3	NA	NA	+		[9]
57	F	IgA lambda	7.44	7.5	11.9	149	+	+	[10]
52	F	IgG kappa	Normal	18.4^*∗*^	Normal	Normal	+		[11]
35	F	IgG kappa	1.66	13.8^*∗*^	NA	NA	+	+	[12]
44	M	Nonsecretory	Elevated	Elevated	NA	NA	+		[13]
42	M	IgG lambda	Normal	Normal	Normal	NA		+	[14]
60	F	Kappa	5	18^*∗*^	11.4	NA	+	+	[15]
52	M	Kappa	2.56	20^*∗*^	5.82	NA		+	[16]
54	F	Nonsecretory	4.9	16.4	7.7	Normal	+		[17]
49	M	NA	7	12.8	NA	NA	+		[18]
51	F	IgG kappa	15.3	12.5	6.6	NA	+	+	[19]
66	F	IgA lambda	2.75	12.8^*∗*^	NA	NA	+		[20]
66	F	IgA lambda	1.92	18.0^*∗*^	NA	NA	+		[20]
47	M	NA	Elevated	Elevated	NA	NA	+		[21]
70	M	IgG	4	17.5^*∗*^	5.2	NA	+		[22]
62	M	IgG	8.6	12^*∗*^	NA	NA	+		[22]
57	M	IgA kappa	7.3	16.7	5.7	NA	+	+	[23]
51	F	IgG	2.01	14.1^*∗*^	NA	NA	+	+	[24]
55	M	IgA lambda	3.1	14.6^*∗*^	3.4	NA	+	+	[25]
53	M	Lambda	3	14.4^*∗*^	4.9	<100	+		[26]
56	M	IgA lambda	3.3	17.5	NA	NA	+		[27]
63	M	NA	NA	16.4^*∗*^	NA	NA			[28]
74	F	IgG lambda	NA	Normal	Normal	Normal			[29]
55	M	NA	NA	Elevated	NA	NA	+		[30]
37	M	NA	4.9	13.1^*∗*^	7.2	NA	+		[31]
73	F	IgG kappa	3.5	15.3	5.8		+		[32]
65	M	IgG kappa	1.8	14.7	4.4	NA		+	[33]
67	M	IgA lambda	4.9	12.5	Normal	NA			[34]
51	M	Kappa	7.01	15.6	6.2	429	+	+	Our case

The blank spaces for “lung lesions on CXR/CT/scintigram or histology finding” and “pathological vessel calcifications” mean that these findings were not described. M: male; F: female; NA: not available. ^*∗*^Not corrected; ^∗∗^findings on CXR/CT/scintigram or histology.
